# Comprehensive Review of the Impact of Maternal Stress on Fetal Development

**DOI:** 10.1002/pdi3.70004

**Published:** 2025-05-10

**Authors:** Divya Tadanki, Pranitha S. Kaza, Elliana Meisinger, Ariana Syed, Asha Johnson, Garen Bainbridge, Michelle Cho, Chikaima Anigbogu, Gargi Gupta

**Affiliations:** ^1^ College of Sciences Georgia Institute of Technology Atlanta Georgia USA

**Keywords:** fetal development, infant and fetal health, maternal mental health, perinatal stress, stress‐induced fetal trauma

## Abstract

Maternal stress impacts millions of pregnant women across the globe each year and refers to mental or psychological stress that occurs during the prenatal period. Although it remains known that maternal stress can negatively impact maternal health, the downstream effects of stress on fetal and infant development in the long‐term are unclear. This leaves a gap in understanding epigenetic factors that impact growth and development, highlighting the need to address potential developmental delays in early childhood. Review of the literature indicates debate over the effect of maternal stress on cognitive development and whether maternal stress is a mediator or causal factor in fetal development. Although evidence suggests that maternal stress disrupts the functional connectivity of the amygdala, information on the sub‐regions of the brain impacted by prenatal stress exposure remains unknown. Studies focusing on environmental disasters and war have also indicated adverse impacts on fetal development, additionally emphasizing a greater need for representation in future research. Timing of fetal exposure to stressors also impacts different domains of development. The Georgia OASIS platform was utilized to investigate variations in reported pregnancies, reported fetal mortality rate, and Perinatal Periods of Risk (PPOR) during the years 2013–2023. Increases in fetal mortality were anticipated to be associated with decreases in the rates of maternal, newborn, and infant care. Although evidence exists to support this hypothesis, causation cannot be established; however, the poor state of maternal healthcare in Georgia warrants further investigation of the barriers to healthcare to improve fetal and infant health outcomes statewide.

## 1 Introduction

For the duration of pregnancy, maternal health plays an important role in both prenatal and postnatal development of the fetus. Maternal stress in particular, is a largely relevant topic of research, as it can directly impact fetal development and lead to the development of long‐term physical, and psychological health issues. Maternal stress encompasses the physical, psychological, and emotional stressors that the mother experiences during pregnancy, these stressors varying in severity and influenced by external environmental factors, socioeconomic status, preexisting physical or psychological conditions, and emotional trauma. According to the existing literature on the subject, this is quantified in research through the self‐reporting of symptoms by the mother, or through the measurement of biomarkers such as maternal endogenous cortisol levels [1]. The objective of this review is to examine existing research and literature on the impact of maternal stress on fetal development, focusing the discussion on biological mechanisms, environmental factors, the potential lasting health impacts, and the gaps in existing literature.

When a stressor is perceived, the hypothalamus activates the stress response regulated by the hypothalamic pituitary and adrenal (HPA) axis by releasing corticotropin‐releasing hormone (CRH). This increase in CRH triggers the pituitary gland to secrete adrenocorticotropic hormone (ACTH). The adrenal cortex is stimulated to release glucocorticoids, mainly cortisol, to activate the sympathetic nervous system [2]. Cortisol is produced as the final product of the HPA axis. It induces the uptake of serotonin, meaning that it can lead to the increased production of serotonin receptors on nerve cells, giving them greater potential to absorb serotonin [3]. Serotonin, or 5‐hydroxytryptamine (5‐HT), is a neurotransmitter responsible for regulating overall emotional well‐being, but it is also an integral part of neuropsychiatric disorders and has a significant impact on brain development. In adults, 5‐HT is unable to cross the blood–brain barrier (BBB), meaning that the brain produces its own serotonin; however, during the first half of pregnancy, 5‐HT can be transferred from the maternal placenta to the fetal brain, establishing a direct link between maternal stress and fetal development [4]. This causes disruptions in the standard development of the fetus, sometimes impacting the gradual growth and physical development, in addition to predisposing the child to mental health disorders. Existing research concludes that abnormal levels of serotonin are associated with mental health disorders, often impacting the developing fetus much later in life [5].

Although valuable research on this topic is existent, a clear consequential relationship between maternal stress and fetal development has yet to be established. Because of the potential for health impacts in the long‐term, this is an area of research that remains relevant and important. The following sections will examine the impact of maternal stress on various aspects of fetal development, including developmental effects and cognitive abilities, molecular changes, physiological development of the fetal brain, developmental programming and environmental influences, as well as the patterns of environmental impact and associated mental health effects.

### 1.1 Developmental Effects and Cognitive Abilities

The findings on cognitive ability have generally conflicted with one another. A study that focused on a population of pregnant women in Chile determined that the timing of stress during pregnancy had a major impact on the child's growth and development [6]. Stress in the third trimester of pregnancy appeared to impact socioemotional behaviors in offspring, whereas stress in the first semester was related to declines in cognition in offspring [6]. This indicates, once again, that the phase of development that the fetus is in plays an important role in determining the effects that maternal stress has in the long‐term. In addition, the sex of the child was also related to the type of developmental change that occurred, with males being more likely to exhibit problems with attention span and females being more likely to exhibit deficiencies in cognitive ability. Given the genetic and developmental changes that vary between the sexes, it follows that there are sex differences in responses to maternal stress. These findings are unique from others in this domain, as they indicate that the period of time in which maternal stress is experienced impacts the developmental outlook. Thus, interventions to protect against stress in these time periods may be useful to protect against adverse effects on the child. The study also indicates that sex, a factor that cannot be controlled for, also influences the development of the child, informing the parents on areas in which they should help their child grow; however, contrasting this finding is a longitudinal study that analyzed the persistence or transience of any changes in offspring due to stress exposure. It was determined that cognitive ability does not change in those exposed to maternal stress, and that any observable behavior changes outside of cognitive ability are not long lasting [7]. This directly contrasts the findings from a year prior, indicating that much remains to be known in this area. The neural changes that arise as a result of stress, such as increased gyrification of the brain, are also correlated with a greater cognitive ability [8]. Although this has not been experimentally confirmed, it is plausible that cognitive ability could be improved in this population. Additionally, a third study found circumstantial evidence of decline in cognitive ability, finding that children exposed to maternal depression generally performed worse on cognitive ability measurement tests, but a strong association could not be verified [9]. This indicates that maternal stress may only serve as a risk factor for adverse child developmental outcomes. Thus, there have been findings that maternal stress either negatively impacts cognitive ability, does not impact cognitive ability, and potentially impacts cognitive ability in a positive manner, or simply serves as a mediating variable that assists with changes in cognition. The findings on the impact of maternal stress on cognitive ability appear to be generally inconclusive and in need of further study.

### 1.2 Changes on a Molecular Level

Building on the understanding of stress and its biological effects, exposure to a stressor triggers the activation of the HPA axis, setting in motion a cascade of physiological responses. Once the stressor has been removed, the cortisol in circulation acts on the paraventricular nucleus (PVN) of the hypothalamus to initiate a negative‐feedback loop that inhibits cortisol production [10]; however, during chronic stress conditions, cortisol levels remain high at all times due to an allostatic shift. Baseline cortisol levels become much higher than normal as the body's HPA axis becomes increasingly sensitive to stressors, causing more cortisol to be released each time when a stressor is encountered [11]. Although constantly high glucocorticoid levels are dangerous to anyone, pregnant women face the most effects. These glucocorticoids are not isolated to only the maternal body, but through the placenta, they are sent to the fetus early in development [12, 13].

Glucocorticoid levels have direct effects on the developing fetus via the placenta. To move through the placenta and into the fetus, the glucocorticoids must overcome the 11β‐hydroxysteroid dehydrogenase type 2 (11 *β*‐HSD2) barrier that acts to protect the fetus from high glucocorticoid concentrations by converting cortisol to its inactive form, cortisone [14, 15]. When this barrier is overcome, fetal exposure to high levels of glucocorticoids can lead to lower birth weights and preterm labor due to decreased 11 *β*‐HSD2 function in the placenta [16, 17]. These low birth rates are consistent with an allostatic shift in the fetus, causing, much like the mother, higher baseline cortisol levels [18]. These higher fasting cortisol levels in the newborn lead to abnormalities in the hypothalamus–pituitary–adrenal (HPA) axis of newborns once they grow into adults. For instance, glucocorticoid receptors (GRs) have altered sensitivity due to constant glucocorticoid exposure. During the development of the hippocampus, exposure to high levels of glucocorticoids can cause changes in DNA methylation in the fetus [19]. This suggests expression of intracellular GRs is altered by epigenetic changes in fetal DNA as chromatin targets containing the *NR3C1* promoter gene (a gene that codes for the expression of glucocorticoid receptors) are methylated, leading to altered stress response later in life [20]. This can be seen with fetal programming, which is the hyperactivity of the HPA axis in female adults who were exposed to high levels of glucocorticoids as fetuses [21, 22].

### 1.3 Physiological Development of the Fetal Brain

Aside from the measurable behavioral changes that occur in children exposed to maternal stress, major regions of the brain, such as the hippocampus and the amygdala, as well as microstructures in the brain, have been the focus of many studies in this field. A study focusing on analyzing the volume of the hippocampus and amygdala in newborns via magnetic resonance imaging (MRI) determined that only the volume of the left hippocampus was markedly lower, whereas the volume of the right hippocampus and the amygdala did not undergo significant change [23]. The relative stability in the amygdala was corroborated by another study that determined that microstructures in the amygdala were relatively unchanged in neonatal brains [24]. This finding is interesting, as the amygdala is involved in the stress response and behavior related to stress, but does not undergo any changes in the fetus; however, connectivity of the amygdala to brain regions such as the prefrontal cortex, the thalamus, and the hypothalamus does change [25, 26]. Thus, although the amygdala itself may not undergo any significant changes due to maternal stress, there appear to be changes in how other brain regions project to the amygdala and how the amygdala projects to the rest of the brain, indicating potential changes in emotional processing ability and regulation. This, in turn, may have downstream effects on the emotional capacity and responses of the child.

The effect of maternal stress on infant brain development is also dependent on whether the baby is born preterm or full term, with preterm babies experiencing a greater change in the functional connectivity of the amygdala to the rest of the brain [26]. This indicates that the developmental stage at which the child is born influences their brain structure and development postnatally, with preterm babies more susceptible to alterations in development. In addition to the role of prematurity, the type of psychological stress also impacts the corresponding changes in neural anatomy and connection. The reduction in hippocampal volume on the left side of the brain and higher degrees of gyrification were observed more often in babies whose mothers had anxiety [8]. Depression, however, resulted in lower creatinine levels in the brain, which relates to alterations in motor ability [8]. The mechanism by which this occurs is unknown, but the current findings indicate that the distinct characteristics of depression and anxiety on a neural level have downstream effects on the development of the child.

A second area of focus in regards to fetal neural development has been microstructures. Although microstructures in the amygdala were unaffected, the microstructures in the right insular cortex were [24]. This suggests a potential impact on socioemotional processing that can lead to the development of anxiety in the offspring, although researchers did not employ a longitudinal study model to observe downstream impacts of changes on the insular cortex. Another change in the limbic system's microstructures was observed in the right uncinate fasciculus [25]. This brain region is involved in processing emotions and socioemotional behavior; however, such conclusions cannot be determined from the study, as it did not analyze observed behaviors later in development.

A third region of study that has been lightly considered is the role of head circumference in fetal development. There is evidence that pregnant women with higher stress levels generally had smaller babies. Higher weight and greater head circumference was positively correlated with improved cognition, providing evidence once again that maternal stress may mediate the development of further cognitive deficits [9]. This is an area that requires further study, however, as literature in this field is lacking. However, this is another argument in favor of an indirect effect of maternal stress on development, rather than a directly causal one [9, 27].

Many of the methodologies used in these studies utilize imaging modalities such as MRI, functional magnetic resonance imaging (fMRI), and diffusion tensor imaging (DTI) to visualize major brain regions, activity in the brain, and changes in microstructures in the brain. Imaging occurred either after birth or in the prenatal period; none of these studies employed a pre‐natal and postnatal scan. These methods are valuable to understand how the fetal brain is changing throughout pregnancy and at various time points after pregnancy; however, many of these studies can only suggest a potential behavioral change that may arise based on neural structures. The lack of prenatal and post‐natal imaging means that the persistence of these changes to neural structures cannot be determined. Thus, although these studies provide valuable insights into the anatomical changes that occur in the brain due to stress, they do not provide observable behavioral evidence of changes that occur as an effect of these changes. Additionally, given that there is general information on the major brain regions impacted by maternal stress, future studies can focus on specific anatomy of these areas to understand the specific neural networks that are impacted and to garner a narrower understanding of the structural changes occurring in the brain.

### 1.4 Developmental Programming

Developmental programming is a term used to describe how environmental factors and other perinatal insults during critical fetal development stages can cause long‐term effects on the health outcomes and risk of diseases/disorders of an individual during their childhood and throughout their adulthood. Perinatal insults such as undernutrition, overnutrition, stress, and other environmental chemicals can have a significant impact on the fetus and may even lead to similar pathological long‐term outcomes due to shared biological methods [28].

Maternal diet is an essential aspect of developmental programming. Maternal undernutrition during pregnancy can increase susceptibility of metabolic diseases and impaired growth and development of internal organs in the fetus, causing permanent long‐term effects on the child [29]. More specifically, undernutrition can also cause intrauterine growth restriction (IUGR), a condition in which the impaired fetal development results in a significantly smaller fetus size and a significantly lower birth weight. IUGR has several implications on fetal growth, such as reduced pancreas size and pancreas beta levels, later leading to inadequate insulin production [30]. This can result in altered metabolic programming and pathway changes which can increase predisposition of conditions such as obesity and type 2 diabetes in offspring [30]. On the other hand, maternal overnutrition during pregnancy can increase chances of macrosomia, where the child is born with excessive weight [29]. This can increase the risk of hypertension and insulin resistance in the offspring in the long term [29].

Furthermore, maternal stress can impact developmental programming as well. Maternal stress during pregnancy can impact the offspring long term in various ways. One of the major ways is the exposure to stress during pregnancy, which can impact developmental programming through increased glucocorticoid levels in the mother and the fetus; this can alter the growth and development of the HPA axis of the fetus, influencing reaction to stress in offspring [31]. Such synthetic glucocorticoids can significantly alter stress responsiveness of children in the long term, and influence neurodevelopmental behaviors which can impact transgenerational health [31].

Maternal stress and chemical exposure can also negatively influence developmental programming in offspring. During neuroendocrine development, gonadotropin releasing hormone (GnRH) is programmed in the fetus. GnRH is a hormone produced in the hypothalamus that stimulates gonadotropin release from the anterior pituitary gland to regulate the reproductive hormone axis. This hormone is significant in influencing sexual development and reproductive functions in males and females; however, perinatal exposure to stress and endocrine disrupting chemicals can negatively impact GnRH neurons [32]. Such exposure can significantly alter *GnRH* gene expression and GnRH neuron morphology, which can disrupt the function of the GnRH hormone and decrease GnRH neuronal presence. This can cause hindered hypothalamic programming in the long‐term and decreased reproductive success in adulthood [32]. Perinatal chemical exposure significantly impacts developmental programming of the fetus, and may cause negative long term side effects in the offspring.

Additional studies have found that maternal stress experienced in the third trimester causes increased infant's susceptibility to developmental programming on the fetus. Such maternal stress can cause premature delivery of infants with immature neurological systems [33]. Furthermore, exposure of prenatal maternal stress (PNMS) during the third trimester can significantly affect the emotional and mental health outcomes in children. This study examined the effect of PNMS induced by a major natural disaster in Quebec, Canada: the 1998 Ice Storm. The study found that maternal stress experienced during the third trimester caused an increase in EAT‐26 scores in children. The EAT‐26 score is a measure of disordered eating in children, and higher scores indicate greater abnormal eating behaviors correlated with exposure to maternal stress in late pregnancy [34]. Developmental programming is also impacted by uteroplacental vascular development, the maturation of blood vessels in the uterus and placenta during pregnancy, which ensures the fetus can obtain adequate nutrients and oxygen and remove all waste. Maternal stress, such as nutrition imbalances, age, and other environmental factors, can impact the proper development of the uteroplacental vascular system, negatively influencing developmental programming and making the fetus more susceptible to long‐term health outcomes such as altered body composition and metabolic function [35].

Overall, maternal stress can negatively impact developmental programming, causing increased susceptibility of the fetus to long‐term chronic conditions such as obesity, metabolic dysfunction, and emotional and mental health outcomes. Studies suggest that developmental programming is most enhanced in the third trimester of pregnancy, also referred to as the “window of vulnerability” during which maternal stress can cause significant implications of developmental programming [34]. Further research into developmental programming can serve to identify additional adverse factors affecting fetal development, and a greater understanding of parental epigenetic inheritance that impacts the influence of developmental programming in the mother and child.

## 2 Patterns of Environmental Impact

### 2.1 Project Ice Storm

Project Ice Storm, a study on a severe ice storm that struck Canada in 1998, used a prospective cohort design to investigate the effects of PNMS on child development. Researchers recruited pregnant women affected by the storm and assessed their stress levels using structured questionnaires and interviews. Outcomes were measured in their children at multiple developmental stages, including birth metrics, cognitive abilities, and behavioral traits, through standardized tests such as the Bayley Scales of Infant Development and parental reports. Infants exposed to higher levels of maternal stress were born with significantly lower birth weights and shorter lengths [36]. These findings highlight the physical sensitivity of fetuses to acute stress during pregnancy. By 30 months of age, children whose mothers experienced heightened stress during pregnancy displayed cognitive deficits, particularly in problem‐solving and attention [36]. This suggests that stress disrupts neural programming during critical developmental windows. Emotional outcomes showed heightened stress reactivity in infants, suggesting that PNMS might alter the development of the autonomic nervous system. This emphasizes the long‐term risk of anxiety and difficulty in emotional regulation. The study concluded that even short‐term acute stressors such as those in Project Ice Storm could significantly impact physical, cognitive, and emotional outcomes, emphasizing the need for interventions that mitigate maternal stress during disasters.

### 2.2 2010 Chile Earthquake

To further explore the impact of environmental stressors, an additional lens is provided by research on the 2010 magnitude 8.8 earthquake in Chile, one of the most powerful disasters recorded in history. Researchers conducted a longitudinal study, tracking pregnant women exposed to the earthquake and comparing them to a control group in unaffected areas. Data collection included clinical measurements of fetal and birth outcomes, such as birth weight, length, and head circumference, as well as interviews to gauge maternal stress levels. The children were followed postnatally to assess neurological and motor development. Infants exposed during the first and second trimesters had significantly smaller head circumferences and shorter body lengths [37]. These results point to the vulnerability of early‐stage fetal organogenesis to stress. Smaller head circumferences correlated with reduced brain volume and potential long‐term cognitive and motor delays. Postnatal follow‐ups suggested impairments in early developmental milestones, such as delayed motor skills and lower cognitive scores. The study also highlighted the long‐term risks of lower birth weights, such as increased susceptibility to metabolic and cardiovascular conditions, aligning with the “thrifty phenotype” hypothesis. The thrifty phenotype hypothesis proposes that poor nutrition or stress during fetal development leads to adaptive changes in the fetus that prioritize immediate survival over long‐term health. These adaptations often involve metabolic programming, where the fetus adjusts its physiology to cope with limited nutrient availability. The researchers concluded that acute stress events such as earthquakes profoundly impact fetal growth and neurodevelopment, with long‐term implications for health and development. They emphasized the importance of timely support systems for pregnant women in disaster zones [37].

### 2.3 2008 Iowa Floods

The 2008 Iowa Floods provided a unique case to study PNMS in a prolonged natural disaster scenario. Researchers recruited pregnant women affected by the flood and assessed maternal stress through cortisol biomarkers, questionnaires, and interviews. Infant health outcomes, including birth weight, immune system function, and stress reactivity, were evaluated through medical records, cortisol levels, and parent‐reported behavioral assessments. Infants born to mothers with higher stress levels had weaker immune systems, evidenced by increased susceptibility to respiratory infections and inflammatory conditions. Elevated cortisol levels during pregnancy likely contributed to these outcomes. Infants displayed heightened stress sensitivity and difficulties with emotional regulation, such as increased reactivity and anxiety‐like behaviors. These behavioral traits were linked to maternal stress‐related disruptions in the fetal autonomic nervous system. Although no significant changes were found in birth weight compared to other disasters, the cumulative stress led to slightly shorter gestation lengths and increased rates of preterm birth. The study concluded that chronic PNMS from disasters such as flooding can have wide‐ranging effects on infant immune and behavioral development, particularly in resource‐limited settings, highlighting the need for community‐based mental health support for pregnant women during prolonged disasters [38].

### 2.4 Sensitivity to Timing of Exposure

Disasters such as that of Project Ice Storm and the Chile Earthquake have shown that exposure during the first trimester has a particularly pronounced effect on physical growth, such as head circumference and birth weight. For instance, babies exposed to the Chile Earthquake in the first trimester were shorter and had smaller head circumferences [37]. First‐trimester exposure appears to impact organogenesis, the critical period of initial organ and brain formation, making this phase exceptionally vulnerable to external stressors. Later‐stage exposure, particularly in the second and third trimesters, has been associated with cognitive, behavioral, and emotional regulation outcomes. Chronic exposure to climate change‐related stressors, such as extreme weather patterns and flooding, occurring closer to birth has been linked to increased rates of preterm birth and emotional reactivity issues in children, highlighting that later exposure can prompt immediate birth complications and long‐term developmental challenges [38]. These insights illustrate that PNMS from disasters such as Project Ice Storm, the Chile Earthquake, and climate change not only affect immediate birth outcomes but can also shape long‐term cognitive, emotional, and health trajectories for the child. This emphasizes the necessity of context‐sensitive and equitable healthcare frameworks that can address the unique needs of pregnant women in diverse ethnic and socio‐economic backgrounds, thereby working to buffer the adverse effects of natural disasters on maternal and fetal health. By recognizing the nuanced impacts of timing and type of disaster exposure, healthcare providers can tailor interventions to support fetal development amidst the growing frequency of natural disasters.

### 2.5 Systemic Challenges in Existing Research

Maternal health is the cornerstone of family and community well‐being, yet it remains disproportionately vulnerable in low‐resource and disaster‐prone areas. More research is urgently needed to understand how maternal stress impacts women in these regions, particularly in the Global South, where poverty, fragile infrastructure, and weak healthcare systems amplify the effects of disasters. Investigating maternal health in such contexts must account for the complex intersections of gender, ethnicity, socioeconomic status, and environmental challenges. Studies should explore how cultural practices, healthcare access, and preexisting health disparities shape maternal stress and, in turn, fetal outcomes. Long‐term maternal health research must extend beyond immediate birth outcomes to include longitudinal studies that track children's physical and cognitive development. This is critical for understanding how systemic inequities during disasters perpetuate cycles of disadvantage across generations. By focusing on maternal health, these studies can illuminate the foundational role mothers play in ensuring resilience and health equity in disaster‐stricken communities [39].

There is also an urgent need for culturally sensitive research methodologies that elevate the voices and experiences of women in underrepresented regions. Partnering with local organizations and stakeholders ensures that research remains relevant and actionable, and that interventions are tailored to the unique challenges these women face. Maternal health cannot be separated from its social and environmental context. Without this focus, interventions will fail to address the root causes of inequities. Focusing on maternal health in disaster research is not only a scientific necessity but an ethical imperative. It is a commitment to ensuring that women and their children, regardless of geography or socioeconomic status, are supported in building healthier futures. Strengthening maternal health systems can mitigate both immediate and long‐term effects of disasters, creating a foundation for resilience and equity across communities worldwide [39].

### 2.6 Generational Trauma

In low‐income countries affected by ongoing conflicts, pregnant women are often subjected to maternal stress, resulting in fetal developmental complications that perpetuate intergenerational trauma [40, 41]. As genocide is impacting the Democratic Republic of Congo, an ongoing study is taking place in order to investigate the result of maternal stress of DNA methylation within newborns and mothers [40]. During the genocide of this country, many pregnant women experienced mental health issues such as post‐trumatic stress disorder (PTSD), manic depression, and anxiety due to the high level of traumatic events. Additionally, while encountering these hardships caused by the war, Congolese women tend to experience socioeconomic and emotional issues due to the lack of access to healthcare, housing, readily available food and water, and the potential separation of families [40]. As a result, maternal fetal attachment (MFA) is contributing to the late development of a newborn's speech, mother and infant interaction, and low birth weight. Not only does the MFA contribute to the mother's stress, traveling to the fetus and creating fetal developmental issues, but cortisol and other stress chemicals also travel through the umbilical cord, where it reaches the fetus and places it in potential danger. In this study, it was determined that the DNA methylation was positively correlated with high levels of maternal stress in Congolese women, primarily in their first or third trimester, experiencing war and generational trauma [40]. These results indicate that these women are often exposed to stressors at the beginning or ending of their pregnancies, causing their DNA to be altered, which can have negative impacts on the fetus. These impacts include slower pace of developing motor skills, gene alterations in the child causing mutations, as well as slow fetal brain development.

Additionally, the alteration of DNA methylation also creates adverse effects within the mothers as the mother's telomeres can be shortened, a decrease of immune system effectiveness, and ultimately death. In addition to this issue of war and generational trauma negatively impacting mother's and fetal development in the Democratic of Republic of Congo, this issue is also occurring in Palestine. Pregnant Palestinian women that reside in Gaza are often triggered by various wars occurring in surrounding areas, causing various mental health issues [41]. Because of the negative emotions the mother is experiencing, the fetus is triggered by the mother's bodily response. This creates an unhealthy relationship between the mother and the baby as pregnancy is considered a time for emotional bonding between the mother and the unborn child. When these stressors are developed through the mother's body, the infant can become detached from the mother, causing an insecure attachment to the fetus and creating a negative relationship between the mother and baby once the child is born.

### 2.7 Intergenerational Transfer

Prenatal stressors not only affect the immediate health of the mother and fetus but can also set the stage for intergenerational trauma, where the consequences of maternal stress ripple through successive generations. Intergenerational transfer refers to observed changes in offspring as a result of physical and biological changes endured by the parents in response to a traumatic event, such as historical, familial trauma, war and conflict, or cultural trauma. The concept of intergenerational transmission relies on the observation that trauma induces long‐lived and widespread effects in the parent. The mechanisms by which traumatic stress is passed onto offspring occur through three primary pathways: the quality of the sperm and egg prior to conception and during genetic transfer, in‐utero complications, and the early postnatal period [42]. These parental biological changes are then “inherited” by the offspring and are reflected in neuroendocrine, epigenetic and neuroanatomical changes. This remains an important concept to understand because it explains how offspring of adult survivors of the Holocaust are at a high risk of developing PTSD, depression, and anxiety and why women who have trauma exposure having to evacuate the World Trade Center on 9/11 give birth to affected offspring [42]. Building on the effects of prenatal stress, the next section delves into its profound impact on maternal mental health and explores its long‐term psychological consequences on fetal health.

## 3 Mental Health Effects

### 3.1 Cortisol and Adrenal Affects

The physiological and psychological stress that expectant mothers experience during pregnancy, known as prenatal stress, has profound implications not only on fetal development but also on maternal mental health, influencing emotional well‐being and long‐term psychological outcomes. The HPA axis mediates stress responses in both the central nervous system and peripheral tissues. During fetal development, the HPA axis is receptive to maternal biological signals. Prenatal stress causes dysregulation of the maternal HPA axis which can lead to chronic elevations of the stress hormone, cortisol. These elevations of cortisol can easily cross the placental barrier, thus exposing the fetus to the stress signals of cortisol which leads to changes in the development of the fetal brain [43]. One such mechanism involves an increased release in proinflammatory cytokines from lymphocytes. These proinflammatory cytokines can cross the placenta and alter the neural circuitry of the fetal brain which in turn can cause behavior changes in adult offspring [44]. A higher risk of schizophrenia in adult offspring is associated with a causal relationship between maternal infection and inflammation. This was shown by exposing pregnant mice to a synthetic analog of double‐stranded RNA called polyriboinosinic‐polyribocytidilic acid (PolyI:C). PolyI:C mimics the acute phase response of viral infection which causes a temporary release of proinflammatory cytokines [45]. This allows for the study of long‐term consequences in offspring born to mothers with increased immune responses and inflammation [46]. This study showed that even a single exposure to PolyI:C in pregnant mice can cause lasting changes in various neurotransmitter levels in the brains of their adult offspring. These altered neurotransmitter levels and overall neurochemical abnormalities in the mice put their offspring at a higher risk for schizophrenia once in adulthood [45]. It is also important to know that there is an increased risk of schizophrenia in offspring born to mothers who experience extreme stress during their first trimester [47].

Maternal stress can manifest in many other ways in offspring. One study identified that maternal anxiety is associated with altered gray matter volume in offspring [48]. Such changes make the offspring vulnerable to psychiatric disorders in addition to cognitive and intellectual impairment. Furthermore, maternal stress has been shown to have a possible linear correlation on impacting fetal brain development due to resulting behavioral problems in offspring of mothers with high anxiety levels. This was identified in a study in which offspring with mothers who had higher levels of anxiety at 32 weeks' gestation were twice as likely to have behavioral and emotional problems by age 4. They noted a strong correlation between prenatal anxiety and offspring's behavioral problems that developed at the age of 4 years. Although this correlation was noted to be independent of gender, there was a higher association of maternal stress with hyperactivity and inattention in boys [49].

The risks of mental disorder, behavioral disturbances, and emotional problems in offspring was shown to be twice as high with mothers who had high levels of anxiety and depression. It is important to note that these effects were shown to be lasting in the offspring, and they were consistent across the ages of 4–13. These findings support an in utero programming hypothesis which states that in‐utero exposure to stress, malnutrition, or other factors can cause lasting effects on the behavior and biology of offspring [50]. Based on the above studies, it is shown that maternal stress has a detrimental, lasting impact on the behavioral, emotional, and cognitive developments of offspring. It is important to take steps to minimize stress during pregnancy to have better outcomes. Relaxation techniques such as yoga, cognitive behavioral therapy, and other interventions can be used to reduce side‐effects of stress in both mothers and infants [51]. Although the adrenal response to stress plays a pivotal role in maternal health, the subsequent hormonal shifts may exacerbate anxiety, a major mental health concern during pregnancy as explored in the next section.

### 3.2 Anxiety

Existing literature examines anxiety in two contexts: anxiety experienced by the mother and the potential development of anxiety in the child later in life. The presence of anxiety in the mother was associated with a reduction in hippocampal volume on the left side of the brain, as well as with greater gyrification [8]. One study utilized psychometrically sound questionnaires to measure the stress levels of expectant mothers. Using MRI, fetal hippocampus volume and frontal and temporal lobe gyrification were compared to the resulting psychological states of the expectant mothers. After exposure to maternal stress in utero, population studies showed an increase in behavioral and neuropsychiatric problems in children, suggesting that maternal anxiety and depression are related to decreased gray matter density and cortical thinning of children. The infants exposed to prenatal maternal anxiety were shown to present slower hippocampal growth, in relation to the left hippocampal volume impact during development [8]. Implications of placental disruptions to fetal development have been considered a mechanism for brain development resulting from maternal anxiety. Maternal psychological distress has been seen to affect the regulation of the HPA axis, causing increased levels of cortisol, which may cause alterations in fetal hippocampus development. Specifically, maternal cortisol levels have been considered to impact the hippocampus and amygdala, which are involved in reactivity and stress regulation. The structural changes are likely the cause of how children's neurological health is directly altered by prenatal stressors. Specifically, the reduction of the hippocampus in the left brain was studied through fetal MRI, with the connection of post‐traumatic stress disorder and schizophrenia being associated with a reduced left hippocampus volume. The increased gyrification in the frontal and temporal lobes from the presence of maternal anxiety has been observed to relate to the increased gyrification index that has been studied in children with autism [8, 52]. Ultimately, the study identifies the role of maternal psychological anxiety and its effect on in‐utero brain development.

Another study indicates that maternal stress corresponds to the development of anxiety, surgency, or negative affect, referring to impulsive behaviors and negatively associated emotions, respectively [27]. One study discussed pre‐ and postnatal anxiety and depression in babies born during the pandemic. Several negative aspects of quarantine were attributed to the decline in maternal psychological health. The results of the study showed higher levels of anxiety and depressive symptoms in women due to the unpredictability of pandemic‐related changes, particularly confinement and difficulties in accessing proper care [27]. The lasting effect of maternal anxiety on offspring was predicted to have a negative impact on the babies' regulation capacities, contributing to issues with emotion regulation. The presenting deficits in children at an early age have been related to more advanced forms in the future, such as depression and anxiety [27]. The particular influences of prenatal health on fetal development were hypothesized to be both genetic and environmental.

Because of the limited scope of study, there is still a large spectrum of unknown effects maternal anxiety may or may not have on offspring. With larger sample sizes and narrowed environmental factors, there is a possibility of finding a standard for the psychological states that affect fetal development. Designation of symptoms to a set generalization could allow for greater accuracy in defining “anxiety” in mothers, allowing for specified research involving the relationship between anxiety levels and fetal development. Further research can be conducted to compare the mechanisms by which maternal anxiety affects the microbiological pathways of in vitro growth with any presenting psychiatric deficits in offspring as they age. Aligning psychological symptoms with biological growth may be the key to understanding how maternal psychological health impacts fetal development.

To explore these trends, the Georgia Online Analytical Statistical Information System (OASIS) platform was utilized to examine variations in reported pregnancies, fetal mortality rates, and perinatal periods of risk (PPOR) from 2013 to 2023. This analysis provides a foundation for understanding the underlying factors influencing these outcomes, which will be further detailed in the following section.

## 4 OASIS Data Trends

**FIGURE 1:**
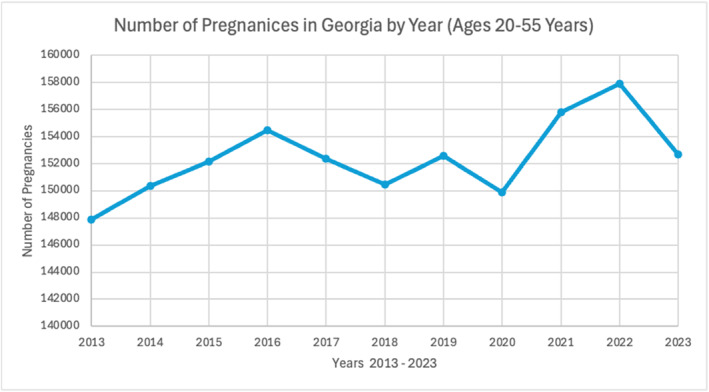
Reported pregnancies of women aged 20–55 years, Georgia, 2013–2023 (Georgia Department of Public Health, Office of Health Indicators for Planning) [54].

The OASIS is a health data repository managed by Georgia's Department of Public Health and accessible to the public. Utilizing this platform, relevant data were extracted to investigate the current state of fetal mortality and maternal health in the state of Georgia. Maternal stress is likely linked to various PPOR. These periods encompass critical stages during pregnancy when maternal health risks, including stress, can significantly impact fetal development, mortality, and overall pregnancy outcomes [53]. By evaluating data from the Georgia OASIS platform, the analysis considers how maternal stress may contribute to these periods of risk, influencing both fetal mortality rates and maternal health during the observed years [54]. Using OASIS, three identified key variables that play large roles in determination of fetal development are pregnancies, fetal deaths, and PPOR, which refers to analyzing both fetal and infant death rates in comparison to maternal/newborn/infant rates [54, 55]. Figure 1 indicates that the number of pregnancies throughout the state of Georgia has remained relatively stable during 2013–2023 [54].

**FIGURE 2:**
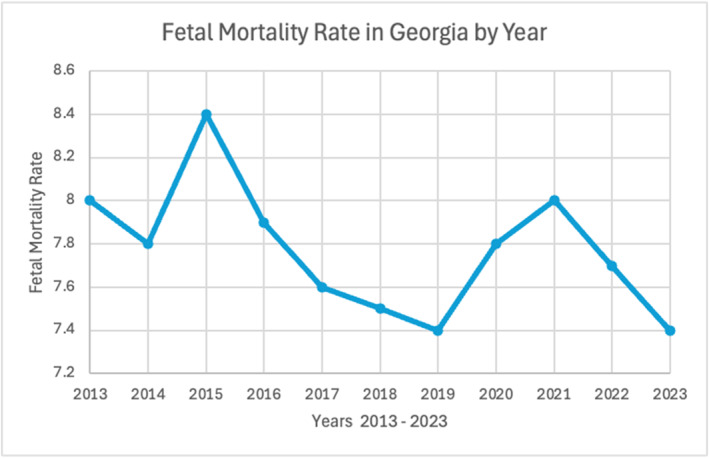
Fetal mortality rate among mothers aged 20–55 years, Georgia, 2013–2023 (Georgia Department of Public Health, Office of Health Indicators for Planning) [54].

The number of fetal deaths and the fetal mortality rate are metrics that may be used in parallel to understand the relationship between fetal survivability and extent of care received specifically in the state of Georgia [56]. Between years 2013 and 2023, the number of fetal deaths and, in conjunction, the fetal mortality rate have decreased (Figure 2).

Data were compiled from OASIS for important factors that affect the fetal mortality rate, such as the rates of newborn care, infant care, and maternal care, as well as the feto‐infant mortality rate, to study relevant trends between the variables. Each of the variables listed are interconnected: the rate of maternal care refers to the frequency of the necessary support received by the mother both during pregnancy and up to 1‐year postpartum, the rate of newborn care refers to the frequency of the necessary support received by the newborn immediately after birth, and the rate of infant care refers to the frequency of the necessary support received by the infant during the first year of life [53, 54].

**FIGURE 3:**
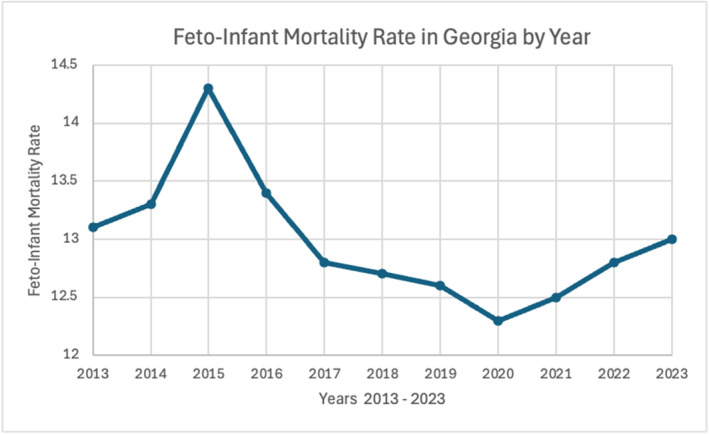
Feto‐infant mortality rate among mothers aged 20–55 years, Georgia, 2013–2023 (Georgia Department of Public Health, Office of Health Indicators for Planning) [54].

**FIGURE 4:**
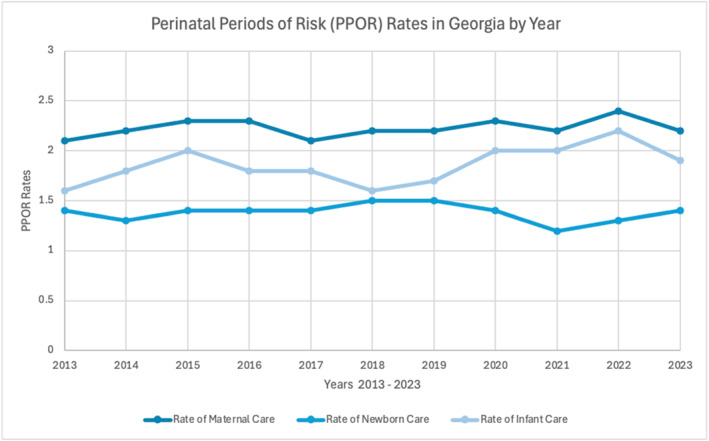
Perinatal periods of risk (PPOR) characterized as the rates of maternal care, newborn care, and infant care among mothers aged 20–55 years, Georgia, 2013–2023 (Georgia Department of Public Health, Office of Health Indicators for Planning) [54].

There has been a decline in the feto‐infant mortality rate (Figure 3) over the past decade alongside an increase in the rate of infant care 2019–2023. Higher rates of maternal care are expected to coincide with higher rates of newborn and infant care, which indicate the use of healthcare services more frequently and thus contribute to lower rates of fetal mortality [54]. Because of simultaneous increases and decreases in the variables, a conclusive relationship of causation cannot be constructed, although the variables are cited to affect each other (Figure 4) [56].

## 5 Discussion

**FIGURE 5:**
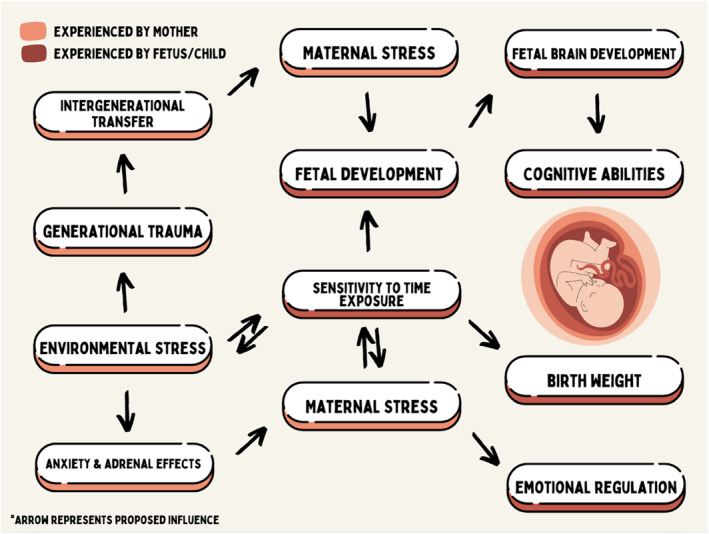
Flowchart depicts the interconnected nature of the proposed factors influencing maternal stress and fetal development.

This review highlights the significant impact of maternal stress on fetal development, emphasizing both the physiological and psychological pathways through which stress exerts its influence. The findings are consistent across existing literature, which associates elevated maternal stress with adverse outcomes such as preterm birth, low birth weight, and altered neurodevelopment in offspring. Stress‐induced dysregulation of the HPA axis emerges as a critical mechanism as elevated cortisol levels associated with chronic stress will cross the placenta and affect fetal development, particularly brain structures such as the hippocampus and amygdala, which play roles in cognition and emotional regulation [57]. Furthermore, stress‐related inflammation appears to exacerbate risks, as increased levels of pro‐inflammatory cytokines may contribute to impair placental function. Epigenetic mechanisms, such as DNA methylation and histone modification, under stress can alter gene expression patterns in the developing fetus, particularly those involved in stress regulation, immunity, and neural development. These epigenetic changes will predispose offspring to chronic health conditions, mental health disorders, and heightened stress sensitivity later in life. This continues to perpetuate a cycle of disadvantage and trauma across generations [53]. Addressing generational trauma and physiological stress require a dual focus: mitigating immediate maternal stress and disrupting the transmission of stress‐induced epigenetic changes. Findings across literature emphasize the importance of early interventions to prevent long‐term intergenerational effects [53]. Additionally, environmental and sociocultural factors modulate the extent of stress‐related fetal effects. Socioeconomic disparities, lack of access to prenatal care, and exposure to discrimination amplify stress levels in marginalized populations. Although a causative relationship cannot be established between healthcare access and positive health outcomes, the trends in feto‐infant mortality rates in Georgia over the past decade highlight that more frequent healthcare usage contributes to lower fetal mortality rates [54]. Figure 5 constructs the complex interplay between biological, psychological, and external factors in shaping fetal development. These findings underline the need for targeted interventions to mitigate stress in vulnerable groups. The review supports the implementation of stress‐reduction strategies during pregnancy, including mindfulness‐based stress reduction (MBSR), cognitive‐behavioral therapy (CBT), and social support programs. MBSR, CBT, and trauma‐informed counseling need to be incorporated into prenatal and postpartum care to help mothers process and manage stress [58].

## 6 Conclusion

Maternal stress is a critical determinant of fetal development, with profound implications for lifelong health and well‐being. This literature review emphasizes the importance of addressing maternal stress through both clinical interventions and broader societal efforts to reduce stressors, particularly in underserved populations. Advocacy for policies that reduce socioeconomic stressors, such as paid parental leave, affordable housing, and equitable access to healthcare, is essential for addressing structural contributors to maternal stress. Public health policies should integrate these approaches into standard prenatal care, prioritizing early identification and intervention for at‐risk individuals. Future research should focus on longitudinal studies to track developmental outcomes into adolescence and adulthood and assess the efficacy of stress mitigation strategies. Reducing maternal stress is not only an investment in maternal health but also in the future health of the next generation.

## Author Contributions

Divya Tadanki: conducted the literature review, thematic designing and ideation, data analysis, and drafting and editing of the overall manuscript. Pranitha S. Kaza: managed project meetings, reviewed literature, thematic designing, data analysis, and drafting and editing of the overall manuscript. Elliana Meisinger: searched literature, reviewed literature, and drafted and edited several sections of the manuscript. Ariana Syed: searched literature, reviewed literature, contributed to data collection and analysis, and drafted data analysis section. Asha Johnson: searched literature, reviewed literature, and drafted few sections of the manuscript. Garen Bainbridge: searched literature, reviewed literature, assisted with data collection, and drafted part of data analysis section. Michelle Cho: searched literature, reviewed literature, assisted with overall editing, and drafted one section. Chikaima Anigbogu: literature search, reviewed literature, and drafted one section. Gargi Gupta: searched literature, reviewed literature, and drafted one smaller section.

## Conflicts of Interest

The authors declare no conflicts of interest.

## Data Availability

The authors have nothing to report.
